# Supplementary material for: Porosity evolution of mafic crystal mush during reactive flow
Source: Nat Commun. 2023 May 29;14:3088. doi: 10.1038/s41467-023-38136-x (PMC10226991; doi:10.1038/s41467-023-38136-x)
Supplement: Supplementary file 1 — Supplementary Information [file 41467_2023_38136_MOESM1_ESM.pdf]

Supplementary Information: Porosity evolution of mafic crystal mush during reactive flow

March 31, 2023

Matthew L. M. Gleeson[1,2*], C. Johan Lissenberg[1], Paula Antoshechkina[3]

[1]School of Earth and Environmental Sciences, Cardiff University, Main Building, Park Place, CF10 3AT, UK.
[2]Department of Earth and Planetary Science, University of California Berkeley, McCone Hall, Berkeley, CA 94720, USA
[3]Division of Geological and Planetary Sciences, Caltech, Pasadena, CA, 91125, USA

*Corresponding author: gleesonm@berkeley.edu

Controls on porosity changes

In the main text, we demonstrate that changes in mush porosity during reactive flow are dominantly controlled by the mineralogy of the dissolved solid assemblage. By considering other variables in the melt-mush reaction models we can also examine why these changes are so critical. Correlation matrices for each scenario modelled (shown in the main text and repeated below) confirm that there is a strong correlation between the Melt Mass Ratio and the proportion of plagioclase and clinopyroxene (or olivine for a troctolitic mush) in the dissolved solid assemblage (Fig. 1). However, these calculations also show that the Melt Mass Ratio is correlated with the mineralogy of the crystallised assemblage (unsurprisingly as this is strongly linked to the mineralogy of the dissolved assemblage) and the change in specific enthalpy of the melt phase (ΔH).

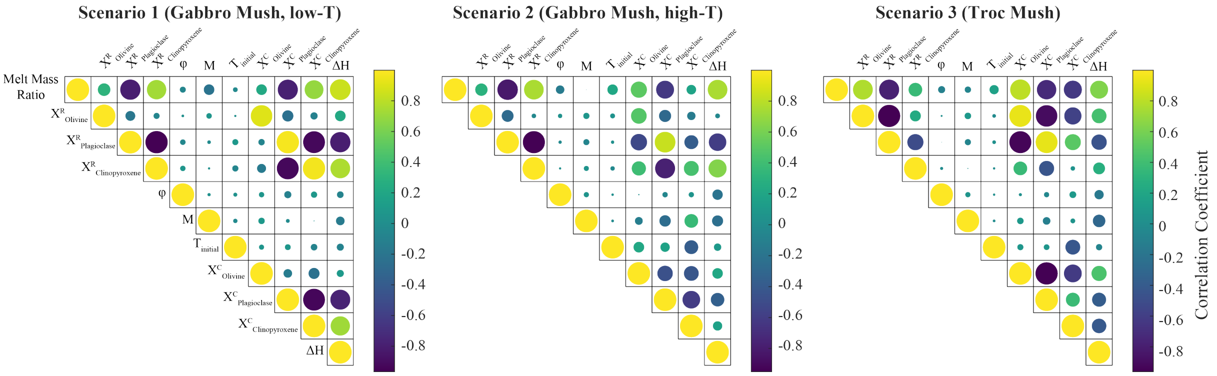

Figure 1: Correlation matrices for each scenario. In all models, there is a strong correlation between the Melt Mass Ratio, the proportion of plagioclase in the dissolved solid assemblage ($X^R_{Plagioclase}$), and the change of specific enthalpy in the melt phase (ΔH defined as the difference between the specific enthalpy of the melt phase prior to, and after melt-mush reaction). There are, however, subtle differences between the scenarios. For example, in scenario 2, there is a weaker correlation between the proportion of clinopyroxene in the crystallised assemblage ($X^C_{Clinopyroxene}$) and the Melt Mass Ratio than there is in scenario 1. This is likely due to the higher temperature of the scenario 2 models and the clinopyroxene-undersaturated nature of the initial melt phase (and thus lower contribution of clinopyroxene to the crystallising assemblage).

In our models, we require that the enthalpy of the entire mush system remains constant (to simulate a thermally equilibrated mush system). As a result, subtle changes in the enthalpy of the melt phase can have significant impacts on the porosity of the mush system. To examine this in more detail, we provide three model runs with plagioclase:clinopyroxene:olivine proportions in the reacted solid assemblage of 75:20:5, 55:40:5, and 25:70:5 (a gabbroic mineralogy is assumed for the wider mush system). Our results indicate that in models dominated by plagioclase dissolution, the difference in enthalpy between the melt phase and dissolved and crystallised solid assemblages increases during melt-mush reaction (Fig. 2). As a result, to maintain the total enthalpy of the mush system, excess crystallisation is required and thus a decrease in the mush porosity. In contrast, when clinopyroxene is dominant, the enthalpy of the melt phase tends towards the enthalpy of the dissolved and crystallised solid assemblages,

meaning that less crystallisation is required to balance the enthalpy of the mush system (and thus an increase in mush porosity).

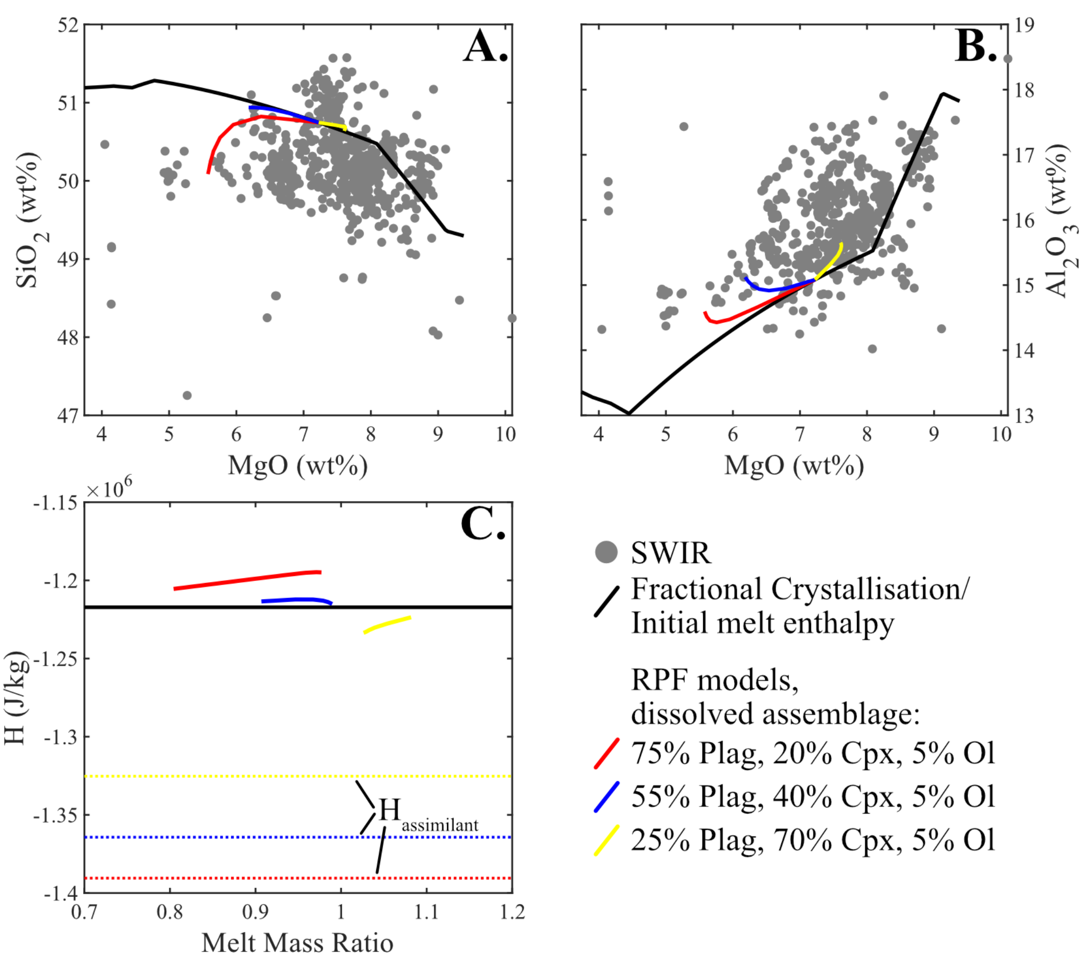

Figure 2: Data shown in **A.** and **B.** represent mid-ocean ridge basalt data of the South-West Indian Ridge from the compilation of Gale et al. (2013). The fractional crystallisation model shown is the same as that used in Fig. 3 of the main text. The reactive flow models initiate at a temperature of 1180 ^{o}C, with an initial mush porosity of 0.2 and M = 0.3. **A.** and **B.** demonstrate that the melt composition during reactive flow deviates from the path defined by fractional crystallisation. **C.** the difference between the enthalpy of the melt phase and the dissolved solid assemblage increases during melt-mush reaction in models where plagioclase forms a dominant component of the dissolved solid assemblage (>50%). This drives excess crystallisation and a decrease in mush porosity.

Mineralogical changes in the mush

One of the key predictions regarding melt-mush reaction in the literature is that is causes a net change in the mineralogy of the affected mush systems. In fact, several studies have proposed that reactive porous flow in gabbroic mush systems might be characterised by a net increase in the modal proportion of clinopyroxene within the mush system (e.g., equation 1 in Lissenberg et al. 2016).

Using our new models we can evaluate the change in the mineralogy of the mush system as a result of reactive flow and melt-mush reaction. Our results indicate that for the base scenario (i.e., porous flow of a three-phase saturated magma through a gabbroic mush system: Scenario 1), we see a net increase in the proportion of clinopyroxene in the mush system at the expense of plagioclase (Fig. 3). In Scenario 2, where the initial melt phase is clinopyroxene undersaturated, a net increase in the modal proportion of plagioclase in the mush is typically observed, potentially aiding the increase in the porosity of the system found in many of these models due to the high latent heat component of plagioclase (Fig. 3). In addition, our results demonstrate that in Scenarios 1 & 3 there is a net increase in the proportion of mafic minerals (olivine + clinopyroxene) relative to plagioclase in the mush. As a result, when the proportion of plagioclase in the reacted assemblage is low (<0.2), little or no plagioclase is expected in the crystallising assemblage, explaining the peak at 0 in panel **H.** of the histograms below.

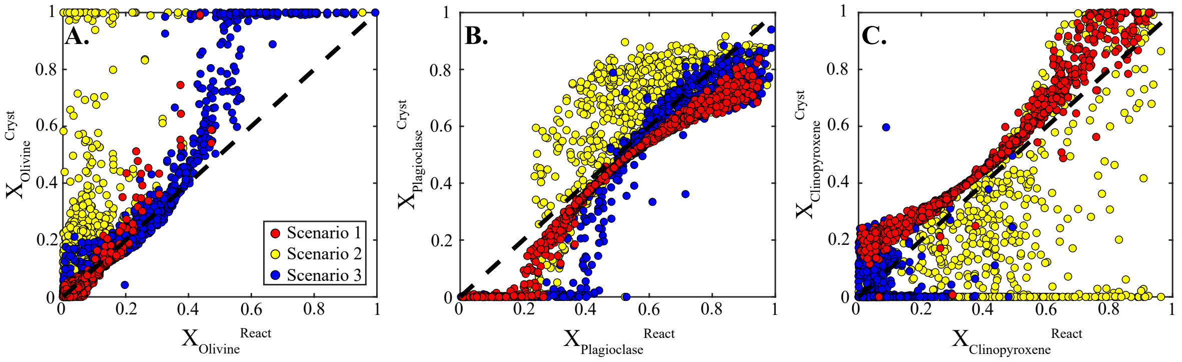

Figure 3: Changes in the mineralogy of the mush, expressed as the proportion of olivine (**A.**), plagioclase (**B.**), and clinopyroxene (**C.**) in the reacted (x-axis) and crystallised (y-axis) assemblage. When data falls above the 1:1 line, this indicates an increase in the proportion of that mineral in the final mush system. For Scenarios 1 & 3, there is a general increase in the proportion of mafic minerals (olivine and clinopyroxene) at the expense of plagioclase in the final mush. In Scenario 2, the clinopyroxene undersaturated nature of the initial melt phases leads to an increase in the proportion of plagioclase in the mush system.

Range of input and output values

Below, we show histograms for all three scenarios modelled in this study that demonstrate the range of input variables (in blue) and outputted crystallisation proportions (in red; Figs. 4, 5), 6).

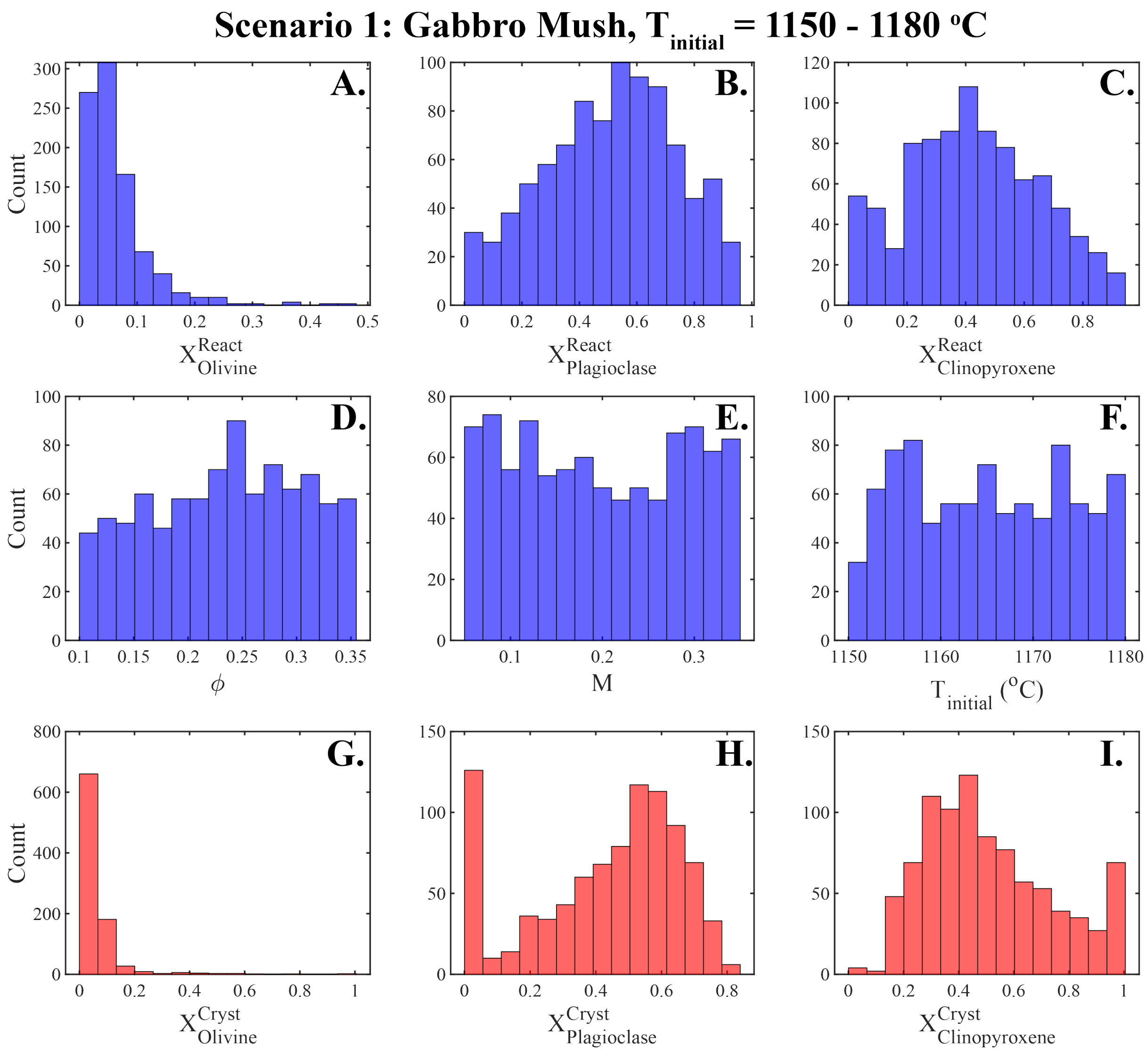

Figure 4: **A. - F.** demonstrate the range of input values used in the 450 simulations (each including 2 steps of melt-mush reaction) displayed in Fig. 3a-c of the main text. **G. - I.** demonstrates the crystallised mineral proportions for these simulations.

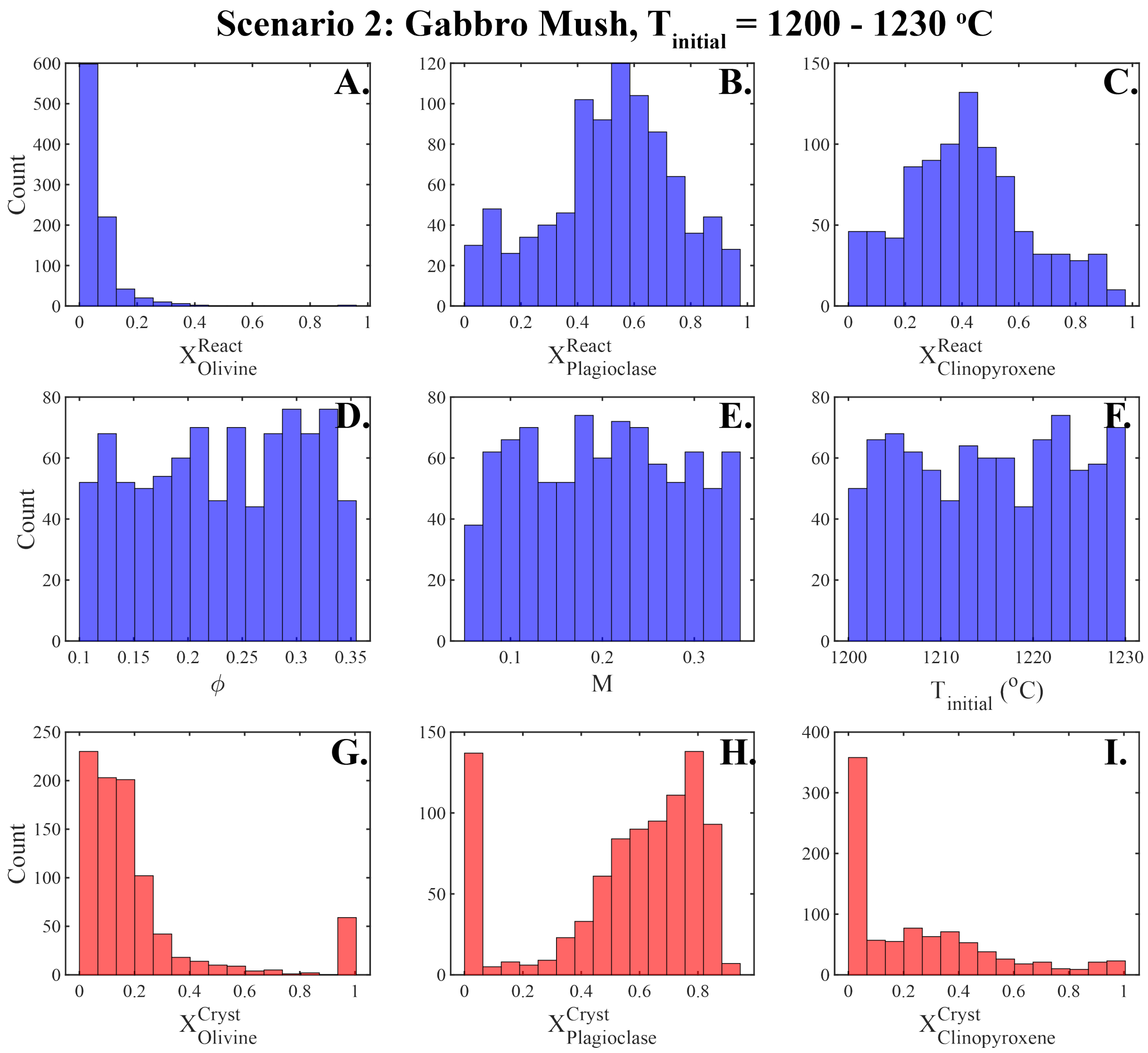

Figure 5: **A. - F.** demonstrate the range of input values used in the 450 simulations (each including 2 steps of melt-mush reaction) displayed in Fig. 3d-f of the main text. **G. - I.** demonstrates the crystallised mineral proportions for these simulations. Notably, compared to Fig. 4, a larger proportion of models show $X^{C}_{Clinopyroxene}$ values <0.2 and $X^{C}_{Plagioclase}$ values >0.6.

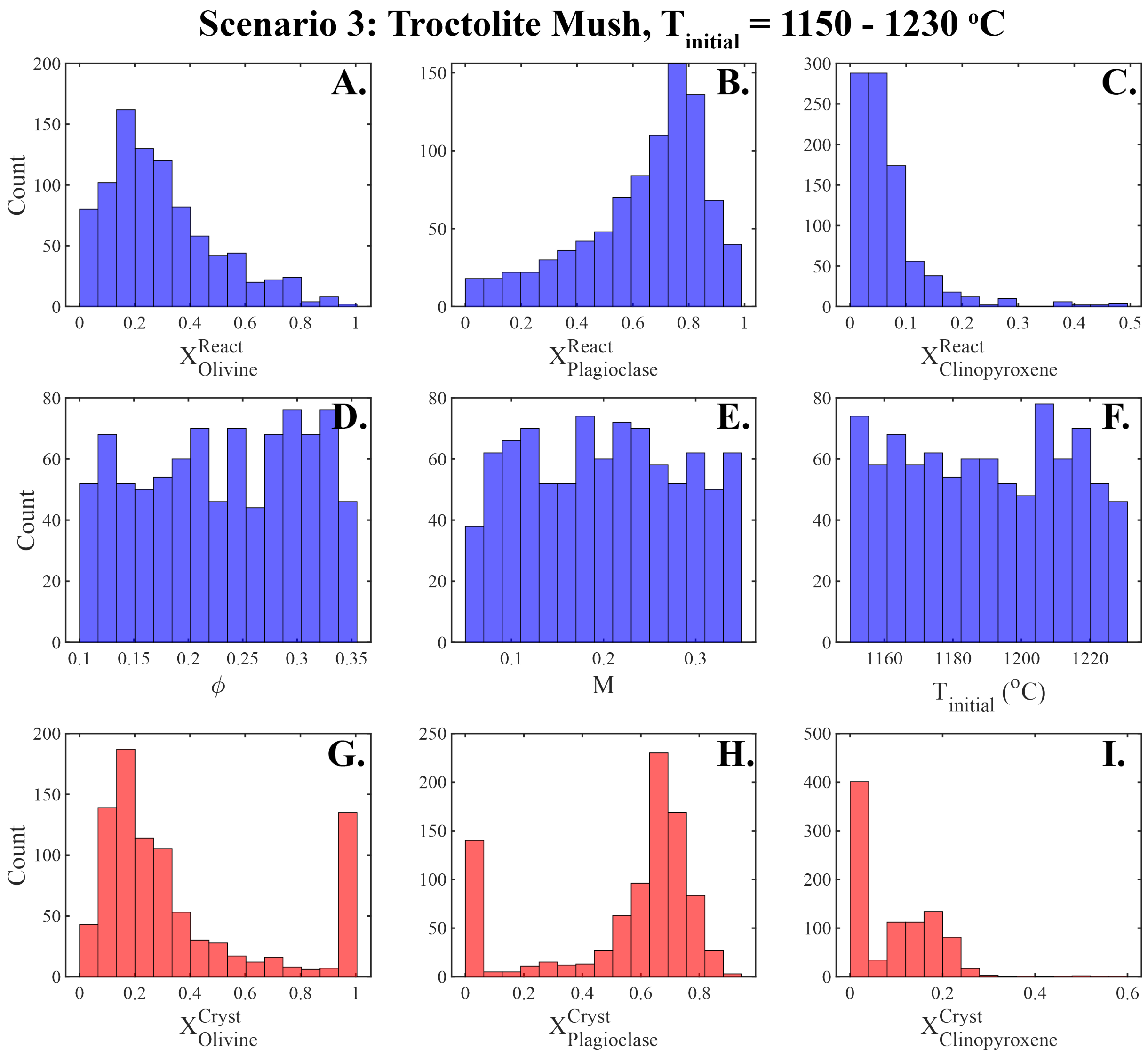

Figure 6: **A. - F.** demonstrate the range of input values used in the 450 simulations (each including 2 steps of melt-mush reaction) displayed in Fig. 3g-i of the main text. **G. - I.** demonstrates the crystallised mineral proportions for these simulations.

Testing different starting compositions

The starting composition used in this study, which is based on the composition of MgO-rich basalts from the South-West Indian Ridge (SWIR) in the compilation of Gale et al. (2013), was chosen for several reasons. First, it represents a reasonable estimate of primary magma compositions beneath the SWIR without having to apply a fractional crystallisation correction. Second, the fractional crystallisation models starting at this proposed primary melt composition provide a good match to the major element data of SWIR basalts, and thus the starting points for our reactive flow/melt-mush reaction models (which are taken from this fractional crystallisation model) are likely appropriate to the SWIR magmas.

Nevertheless, the choice of starting composition could, potentially, have an influence on our model results. To examine this possibility we re-ran all Scenario 1 & 3 models with the melt starting composition set to that of the most primitive Fracture Zone basalt from Dick et al. (2000). Models were not re-run for Scenario 2 as the liquidus temperature of this basalt at the pressure of the models (100 MPa) is only $\sim 1205^{o}$C. Additionally, only Scenario 3 models with a starting temperature below this liquidus temperature were used in the following figures. Overall, we can see that the choice of starting composition has very little influence on the results of our models (Figs. 7, 8, & 9).

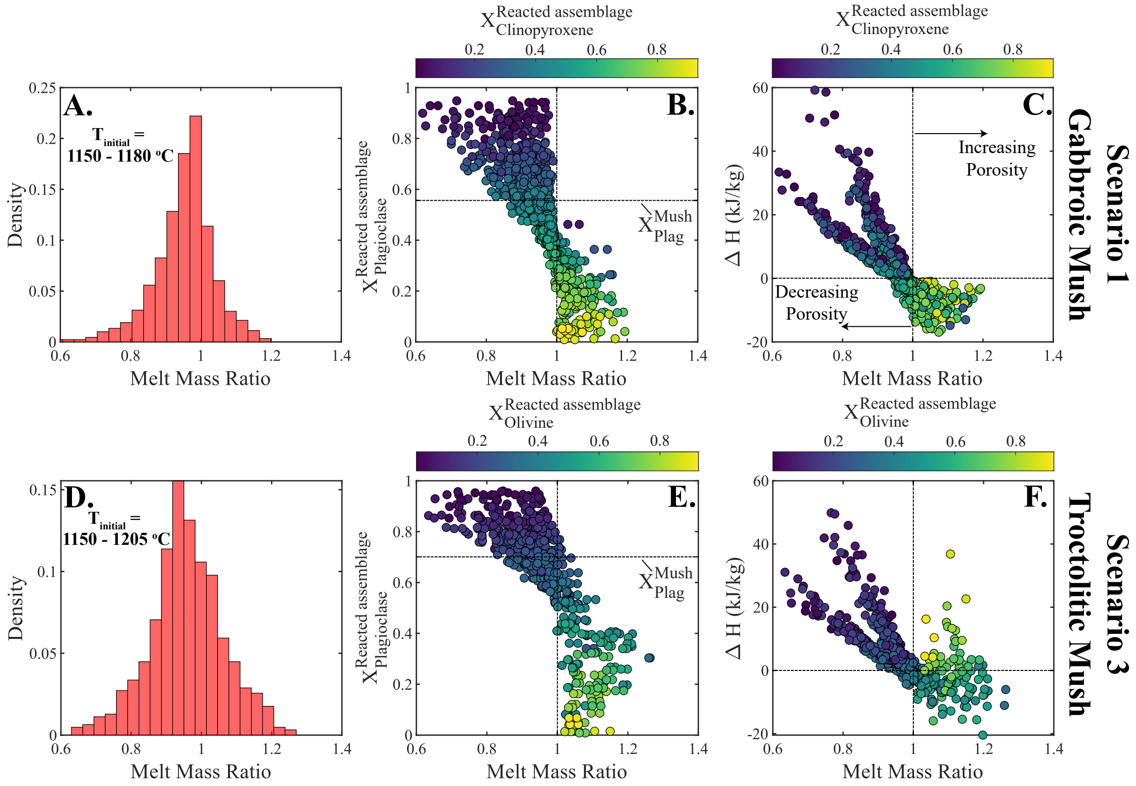

Figure 7: Melt-mush reaction model results for calculations using a starting melt composition from Dick et al. (2000). Scenario 1 (**A. - C.**) & 3 (**D. - F.**) models are shown, and display very similar results to Fig. 3 of the main text.

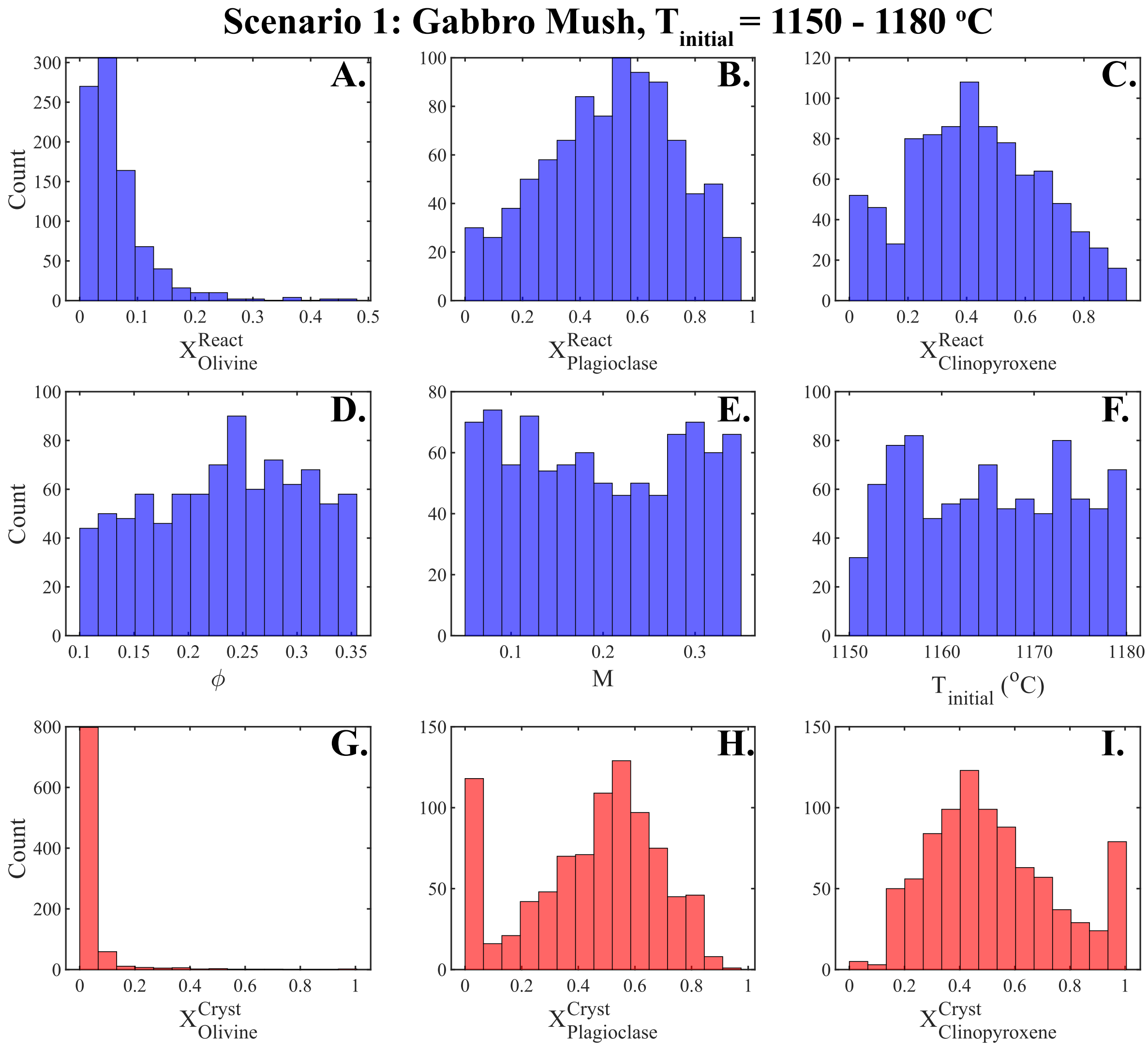

Figure 8: **A. - F.** demonstrate the range of input values used in the 450 simulations (each including 2 steps of melt-mush reaction) displayed in Fig. 7 panels **A. - C.**. **G. - I.** demonstrates the crystallised mineral proportions for these simulations.

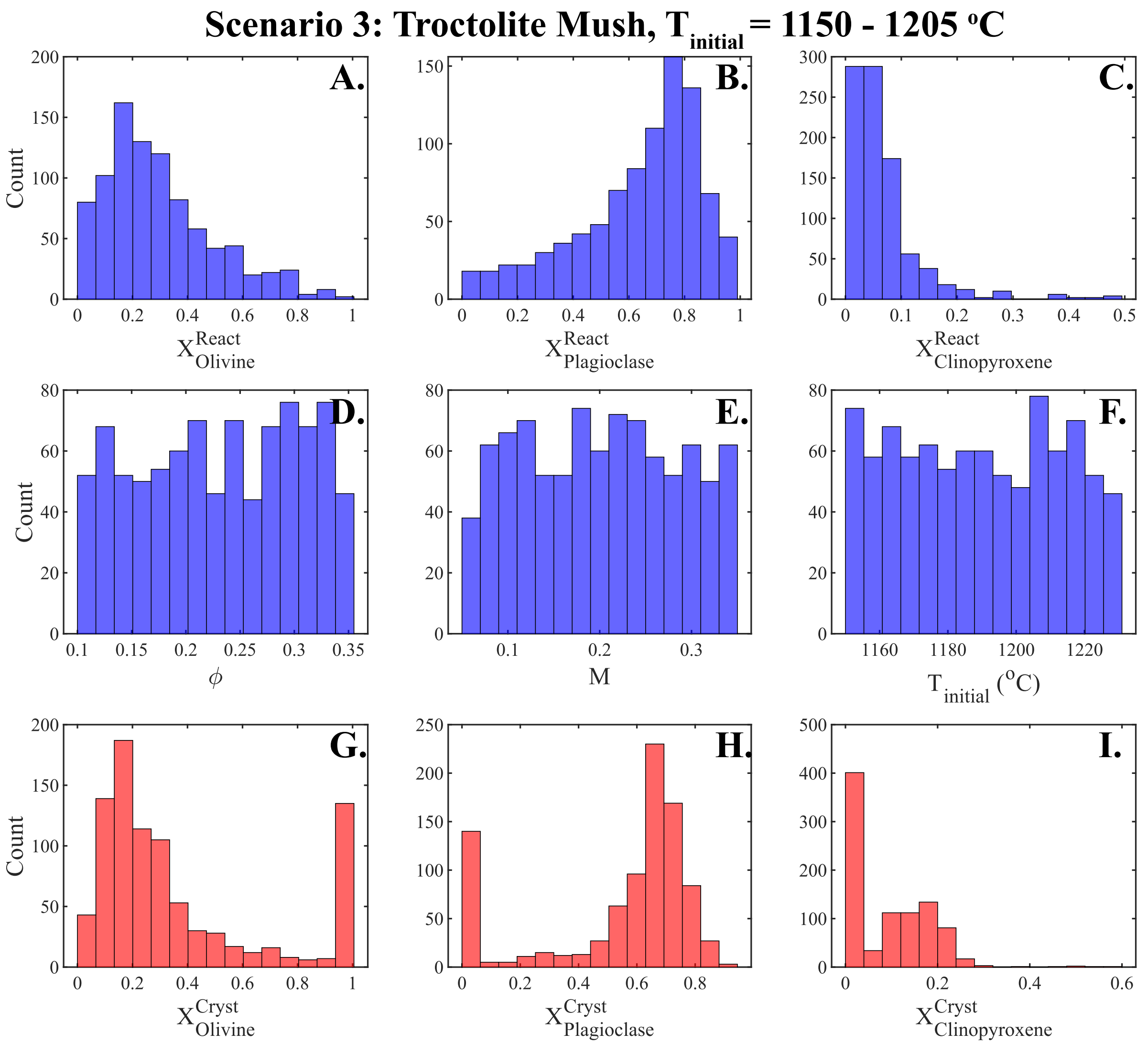

Figure 9: **A. - F.** demonstrate the range of input values used in the 450 simulations (each including 2 steps of melt-mush reaction) displayed in Fig. 7 panels **D. - F.**. **G. - I.** demonstrates the crystallised mineral proportions for these simulations.

References

1. Lissenberg, C.J. and MacLeod, C.J., 2016. A reactive porous flow control on mid-ocean ridge magmatic evolution. Journal of Petrology, 57(11-12), pp.2195-2220.
2. Gale, A., Dalton, C.A., Langmuir, C.H., Su, Y. and Schilling, J.G., 2013. The mean composition of ocean ridge basalts. Geochemistry, Geophysics, Geosystems, 14(3), pp.489-518.
3. Dick, H.J., Natland, J.H., Alt, J.C., Bach, W., Bideau, D., Gee, J.S., Haggas, S., Hertogen, J.G., Hirth, G., Holm, P.M. and Ildefonse, B., 2000. A long in situ section of the lower ocean crust: results of ODP Leg 176 drilling at the Southwest Indian Ridge. Earth and planetary science letters, 179(1), pp.31-51.
